# Expression Profile and Clinical Relevance of ADAR Family Genes in Head and Neck Squamous Cell Carcinoma

**DOI:** 10.3390/genes16111316

**Published:** 2025-11-02

**Authors:** Tomasz Kolenda, Piotr Białas, Paulina Poter, Marlena Janiczek-Polewska, Anna Zapłata, Kacper Guglas, Patrycja Mantaj, Anna Przybyła, Urszula Kazimierczak, Ewa Leporowska, Zefiryn Cybulski, Anna Teresiak

**Affiliations:** 1 Research and Implementation Unit, Greater Poland Cancer Centre, Garbary 15, 61-866 Poznan, Poland; zefiryn.cybulski@wco.pl (Z.C.); anna.teresiak@wco.pl (A.T.); 2 Microbiology Laboratory, Greater Poland Cancer Centre, Garbary Street 15, 61-866 Poznan, Poland; 3 Department of Cell Biology, Poznan University of Medical Sciences, Rokietnicka 5D, 60-806 Poznan, Poland; 4 Laboratory of Cancer Genetics, Greater Poland Cancer Centre, 15 Garbary Street, 61-866 Poznan, Poland; paulina.poter@wco.pl (P.P.); kacper.guglas@wco.pl (K.G.); annaprzybyla@ump.edu.pl (A.P.); 5 Department of Oncologic Pathology and Prophylaxis, Greater Poland Cancer Centre, Poznan University of Medical Sciences, 15 Garbary, 61-866 Poznan, Poland; 6 Department of Clinical Oncology, Greater Poland Cancer Centre, Garbary 15, 61-866 Poznan, Poland; marlena.janiczek@wco.pl; 7 Department of Electroradiology, Poznan University of Medical Sciences, Garbary 15, 61-866 Poznan, Poland; 8 Department of Laboratory Diagnostics, Greater Poland Cancer Centre, Garbary 15, 61-866 Poznan, Poland; anna.zaplata@wco.pl (A.Z.); ewa.leporowska@wco.pl (E.L.); 9 Radiation Protection Department, Greater Poland Cancer Centre, Garbary 15, 61-866 Poznan, Poland; patrycja.mantaj@wco.pl; 10 Department of Diagnostics and Cancer Immunology, Greater Poland Cancer Centre, Garbary 15, 61-866 Poznan, Poland; ukazimierczak@ump.edu.pl; 11 Department of Cancer Immunology, Poznan University of Medical Sciences, Rokietnicka 8, 60-806 Poznan, Poland

**Keywords:** ADAR1, ADAR2, ADAR3, deaminase adenosine, RNA metabolism, head and neck cancers, TCGA, biomarker

## Abstract

**Background:** ADAR1 (*ADAR*), ADAR2 (*ADARB1*), and ADAR3 (*ADARB2*) are deaminase adenosine RNA-specific enzymes that play a significant role in RNA metabolism. ADAR1 (*ADAR*) and ADAR2 (*ADARB1*) catalyze A-to-I editing and ADAR3 (*ADARB2*) plays a regulatory role. The role of these three genes still remains unknown in head and neck cancers (HNSCC). The aim of this study is to reveal the role of deaminase adenosine RNA-specific enzymes in pathomechanisms of HNSCC and to investigate their potential utility as diagnostic and/or prognostic biomarkers. **Methods:** The quantitative PCR analysis was conducted using RNA isolated from 22 pairs of matched tumor and adjacent normal tissues, 76 formalin-fixed paraffin-embedded (FFPE) tumor samples, and a panel of HNSCC cell lines (DOK, SCC-25, SCC-40, FaDu, and CAL-27). In parallel, transcriptomic and clinical data from the Cancer Genome Atlas HNSCC cohort were analyzed. Patients were stratified into high- and low-expression groups, and statistical assessments included overall survival and progression-free interval analyses, evaluation of gene expression in relation to clinicopathological parameters, correlation with other genes, and functional pathway exploration using gene set enrichment analysis. **Results:** ADARB2 was significantly downregulated in HNSCC tumor tissues compared to adjacent normal mucosa (*p* = 0.044), with discriminatory potential to distinguish malignant from non-malignant tissues (AUC = 0.692, *p* = 0.029). TCGA data confirmed *ADAR* (*p* < 0.0001) and *ADARB1* (*p* < 0.0001) upregulation in tumors, while *ADARB2* was markedly reduced (*p* = 0.04). Patients with high *ADARB2* expression showed significantly longer overall survival (pa = 0.0121; pb = 0.0098), with a trend toward improved progression-free survival (pb = 0.0681). Subsite analysis revealed high *ADAR* expression correlated with poor OS in pharyngeal tumors (*p* < 0.05), whereas high *ADARB2* expression was linked to improved DFS (pa = 0.0023, pb = 0.0047). GSEA indicated that low *ADARB2* expression was enriched in oncogenic pathways, including Wnt/β-catenin (*p* = 0.006), MYC targets (*p* = 0.009), and TGF-β1 (*p* = 0.009). **Conclusions:** *ADARB2* expression was significantly reduced in HNSCC tumor tissues compared to normal mucosa and demonstrated strong discriminatory power for distinguishing malignant from non-malignant samples. High *ADARB2* expression was associated with markedly improved overall survival, whereas low expression correlated with enrichment of oncogenic pathways, including Wnt/β-catenin, Notch, and Hedgehog, consistent with a poorer clinical prognosis. These findings highlight *ADARB2* as a promising diagnostic biomarker and independent prognostic factor in HNSCC.

## 1. Introduction

Head and Neck Cancers (HNCs) represent a heterogeneous group of malignancies that originate in various anatomical sites within a single region. This is a broad term that includes tumors arising in the upper gastrointestinal tract, e.g., paranasal sinuses, nasal cavity, oral cavity, pharynx and larynx, as well as tumors arising from the salivary glands, thyroid and parathyroid glands. The most common histological subtype is Head and Neck Squamous Cell Carcinoma (HNSCC), which accounts for over 90% of all head and neck cancer cases [1,2]. These tumors have been assigned to basic groups due to location, clinical and biological features. According to the U.S. National Institutes of Health (NIH), primary categories include cancers of the oral cavity, pharynx, and larynx.

Globally, HNCs are the fifth most common cancer type, with an incidence ranging from 0.5 to 43.1 cases per 100,000 individuals [3]. According to the study performed by Makowski et al. in the groups of ages 60–69, 70–79, and 80+ the increasing number of incidents of HNSCC and mortality is observed in the Polish population [4]. It should be noted that upward trends until 2035 underline the need for better prevention and treatment strategies [5].

Head and neck cancers, like other cancers, are most often caused by many factors (diet, social and cultural habits, genetic background), however the most important risk factor for HNC is smoking and excessive alcohol consumption [6,7]. Both of these factors have a synergistic effect [7]. Tobacco smokers are up to six times more likely to develop head and neck cancer than non-smoker individuals, and 20–30% of oral cancers are closely related to smoking or chewing tobacco [8,9]. Alcohol, on the other hand, is directly responsible for 19% of all oral cancers in the world [9]. Furthermore, excessive chronic alcohol consumption increases the risk of cancer of the upper gastrointestinal tract by about 2–3 times [10].

An important exogenous risk factor for HNCs is infection with high-risk types of human papillomavirus (HPV), particularly HPV16 and HPV18. These viral genotypes are implicated in 25–30% of oropharyngeal squamous cell carcinomas, with HPV16 alone accounting for over 90% of HPV-positive cases [11,12]. The recognition of HPV as an etiological factor has led to the subclassification of HNCs into HPV-positive and HPV-negative tumors, which differ significantly in terms of molecular features, epidemiology, clinical progression, and treatment outcomes. Furthermore, it should be highlighted that they comprise a highly heterogeneous group of tumors, which complicates accurate prognosis and personalized therapy [13].

Histologically, HNSCCs can be divided into multiple subtypes, and molecular classification based on transcriptomic analysis has further refined this categorization. According to The Cancer Genome Atlas (TCGA), four molecular subtypes have been identified: atypical, mesenchymal, basal, and classical [14]. Additionally, HPV status introduces another layer of heterogeneity, reflecting distinct mutational landscapes and alterations in pathways [13]. As part of the TCGA initiative, data from 279 HNSCC patients were analyzed, revealing frequent genomic aberrations and disrupted signaling networks associated with carcinogenesis [14,15]. Common genetic events in HNSCC include deletions of chromosomal regions 3p and 9p, inactivating mutations in *TP53*, loss of *CDKN2A*, and amplification of *CCND1*, resulting in the dysregulation of the G1/S cell cycle checkpoint [14,16]. The overexpression of cyclin D1, coupled with the inactivation of p53, contributes to unchecked proliferation and impaired apoptosis. Furthermore, growth factor receptors, such as EGFR and MET, are often overexpressed or amplified, further promoting oncogenic signaling [15].

Significant alterations have also been observed in the Wnt signaling pathway. The *FAT1* encodes a cadherin-like protein that is involved in cell adhesion and polarity and is mutated in approximately 23% and deleted in 8% of HNSCC cases [17]. Another altered gene, *AJUBA*, encodes a LIM-domain protein associated with mitotic regulation and cellular adhesion [18]. Moreover, mutations in *NOTCH1*, a gene typically acting as a tumor suppressor, are also prevalent, although its precise role in HNSCC remains under investigation [15]. In HPV-positive tumors, carcinogenesis is primarily driven by the viral oncogenes E6 and E7, which inactivate tumor suppressors p53 and RB1, respectively [19]. Importantly, in these tumors, the genes encoding p53 and RB1 are usually not mutated, as their functions are directly impaired by viral proteins. A characteristic molecular feature of HPV-driven HNSCC is the activation of the PI3K/AKT/mTOR pathway, which promotes cell survival, proliferation, and migration [15].

Clinically, HPV-positive tumors tend to be smaller in size but are more likely to present with advanced lymph node metastases. Despite this, they are associated with a more favorable prognosis and a better response to radiotherapy and chemotherapy [20,21,22]. The epidemiological profile of these patients is also distinct: they are typically younger, non-smoking white males [23]. The management of HNCs involves both surgical and non-surgical approaches. Surgical treatment remains the cornerstone of therapy and involves tumor excision with clear margins and, if necessary, regional lymph node dissection [24]. Given the anatomical complexity of the head and neck region, surgeries are designed to minimize functional impairments in breathing, speech, and swallowing. In some cases, reconstructive surgery is performed to restore anatomical integrity [25]. Adjuvant therapies, including radiotherapy, chemotherapy, and immunotherapy, are proposed mainly based on tumor stage, location, molecular profile, and patient health status [26,27,28,29,30]. Consequently, transcriptomic profiling to identify gene expression changes offers new opportunities to enhance the efficacy of radio- and chemotherapy [31,32,33]. In the case of HPV-positive tumors, nanotechnology is considered a promising application in cancer immunotherapy [34,35]. The use of new techniques and the creation of appropriate panels consisting of molecular biomarkers seems to be an approach that will allow for improving the standards of therapy [36,37,38].

At the molecular level, post-transcriptional regulation of gene expression via adenosine-to-inosine (A-to-I) RNA editing has emerged as a crucial mechanism in both normal physiology and tumor biology. This process is catalyzed by enzymes of the adenosine deaminase acting on the RNA (ADAR) family [39,40]. A-to-I editing can result in codon changes, alternative splicing, and modulation of miRNA processing, thereby influencing transcriptome and proteome diversity [41]. Dysregulation of this editing mechanism contributes to cancer progression, as it can alter the expression and function of critical genes.

The ADAR family includes three members: ADAR1 (encoded by *ADAR*), ADAR2 (encoded by *ADARB1*), both of which have enzymatic activity, and ADAR3 (encoded by *ADARB2*), which lacks catalytic function and appears to act as a regulatory protein [42,43,44]. ADAR1 and ADAR2 are ubiquitously expressed, whereas ADAR3 expression is largely restricted to the brain [45]. ADAR1 is frequently overexpressed in various malignancies, including hepatocellular carcinoma, esophageal squamous cell carcinoma, and breast cancer. One well-characterized substrate is AZIN1, whose edited form produces a protein variant that resists degradation and promotes oncogenicity by stabilizing ornithine decarboxylase and cyclin D1, facilitating increased proliferation and tumorigenic potential [46,47,48,49]. In thyroid cancer, high ADAR1 expression correlates with worse prognosis, while loss of catalytic activity impairs tumor formation and cell proliferation in 3D culture models [50]. ADAR1 also modulates miRNA maturation, including the negative regulation of let-7d, which affects self-renewal in chronic myeloid leukemia [51]. ADAR2 is best known for its role in editing the GluR-B subunit of the glutamate receptor, and its dysfunction is associated with neurological disorders and malignancies [52,53,54]. In gliomas, reduced ADAR2 activity is associated with lower levels of CDC14B, a phosphatase involved in the degradation of SKP2, which, in turn, regulates p21 and p27, two key cell cycle inhibitors [55,56]. Thus, loss of ADAR2 leads to increased SKP2 activity and cell cycle progression. ADAR2 also responds to dietary and metabolic cues, such as high-fat diets, where it influences insulin secretion in pancreatic β-cells, suggesting a dual role in metabolism and tumor suppression [57,58]. ADAR3, while lacking RNA-editing activity, may still have biological relevance. Its expression is predominantly neuronal, and though its role in cancer is not well defined, high levels of ADAR3 (*ADARB2*) correlate with improved prognosis in lower-grade gliomas (LGG), suggesting a possible tumor-suppressive function in specific contexts [59]. Despite accumulating evidence implicating ADAR family enzymes in carcinogenesis, their role in HNSCC remains largely unexplored.

This study addresses this gap by systematically evaluating *ADAR*, *ADARB1*, and *ADARB2* expression in cell lines, patient samples, and TCGA cohorts.

## 2. Materials and Methods

### 2.1. Cell Lines

Commercially available cell lines from the head and neck area were used for experimental analysis: DOK, SCC-25, SCC-40, FaDu and CAL-27. The cells were cultured under routine conditions (37 °C; 5% CO_2_) in dedicated media with FBS (10% *v*/*v*, BioWest, Nuaillé, France) and gentamicin (20 mg/mL, Kirk, Poland).

### 2.2. Patient Material

The total number of analyzed cases was 98. This group consisted of 22 cases obtained during surgical procedures; however, only 20 of these were available for RNA isolation and evaluation of gene expression. In addition, 76 cases were archival material originating from previous studies [60].

The examined tissues were obtained from patients with HNSCCs during surgical procedures at the Greater Poland Oncology Centre (Poland) in 2010–2011. The samples consisted of 22 pairs of slides from patients, including neoplastic tissue and non-cancerous epithelial tissue (collected with a minimum of 2 cm distance from the border of the tumor). Prior to tissue collection, none of the patients underwent preoperative chemo- or radiotherapy. Neither was diagnosed with a local recurrence or second primary tumor. Histopathological examination confirmed that all tissues were classified as HNSCCs, and their HPV infection status was negative. The tumor cell differentiation grade was determined according to the World Health Organization guidelines, and the TNM classification was noted in accordance with the recommendations of the Union for International Cancer Control [61,62]. Moreover, the HPV infection status of the tissue samples was determined using p16INK4A (p16) immunohistochemistry (IHC) CINtec^®^ p16 Histology (Roche Diagnostics; Indianapolis, IN, USA) antibody according to the manufacturer’s protocol.

### 2.3. Database

The study used publicly available data that was made available as part of the TCGA (The Cancer Genome Atlas) project. Gene influence data and pictures of disease effects with HNSCCs were retrieved from the University of California, Santa Cruz website: (https://xenabrowser.net/datapages/; accessed on 1 March 2020) for 515 cases. Genetic gene correlations were retrieved from the cBioportal For Cancer Genomics website (www.cbioportal.org; accessed on 1 March 2020). Data on the expression of the studied genes in 24 different types of cancers, non-cancerous tissues (44 cases), and cancer tissues (520 cases) were retrieved from the UALCAN database (https://ualcan.path.uab.edu/; accessed on 1 March 2020).

### 2.4. Analysis of ADAR, ADARB1, and ADARB2 Expression

RNA Isolation

RNA isolation from cell lines was performed using the GeneMATRIX Universal RNA/miRNA Purification Kit (EURX) according to the manufacturer’s protocol.

RNA isolation from 20 non-cancerous and neoplastic tissues was performed using TRI reagent (Sigma-Aldrich, St. Louis, MO, USA) according to SOP (standard operating protocol). To concentrate the RNA and remove DNA contamination, the High Pure miRNA Isolation Kit (Roche, Basel, Switzerland) was applied. Archival Formalin-Fixed Paraffin-Embedded (FFPE) tissues were prepared by the Department of Cancer Pathology, Greater Poland Oncology Centre, and delivered as 10-µm sections. After paraffin removal, RNA was isolated using the High Pure FFPET Isolation Kit (Roche) according to SOP.

Reverse transcription reaction

In order to obtain cDNA, templated RNA (0.5 μg) incorporation, reverse transcription was performed using the iScript cDNA Synthesis Kit (Bio-Rad, Hercules, CA, USA). The reaction was conducted according to the SOP provided by the Kit producer.

Primers design and Real-time PCR

Primers for *ADAR* and *ADARB1* were adapted from Altaf et al. [61], while *ADARB2* primers were designed using the Primer3Plus tool (version 2.3.7.) and validated with the NCBI BLAST platform. All primers were synthesized by Genomed (Warsaw, Poland). Relative gene expression was normalized to 18S rRNA using the 2^−ΔCT^ method.

Gene expression levels of *ADAR*, *ADARB1*, and *ADARB2* were quantified using SYBR Green-based real-time PCR. Specific primers and the 2x SYBR Green Master Mix (Roche) were used in the reactions.

RNA concentration and purity were assessed using a NanoDrop 2000 spectrophotometer (Thermo Fisher Scientific, Waltham, MA, USA). RNA integrity was verified by evaluating the 28S and 18S rRNA bands through native agarose gel electrophoresis (1%), similar to what was described earlier [63].

### 2.5. Pathological and Clinical Analysis

Clinical data were analyzed using the log-rank (Mantel–Cox) and Gehan–Breslow–Wilcoxon tests to assess differences in progression-free survival. Patients were stratified by the following clinical parameters: age (<60 vs. ≥60), sex (female vs. male), alcohol use (yes vs. no), smoking status (yes vs. no), tumor differentiation grade (G1 + G2 vs. G3 + G4), tumor size (T1 + T2 vs. T3 + T4), lymph node involvement (N0 vs. N1–N3), perineural invasion (yes vs. no), cancer stage (I + II vs. III + IV), cervical lymphadenectomy (yes vs. no), and HPV status (positive vs. negative).

Smokers were classified following NIH categories: current smokers and those who quit less than 15 years ago (categories 2 and 4) were grouped as “smokers,” while never-smokers and those who quit more than 15 years ago (categories 1 and 3) were grouped as “non-smokers” [64].

Comparisons between groups were performed using Student’s *t*-test or the Mann–Whitney U test, depending on data distribution (assessed by normality tests).

Genes with Spearman’s correlation coefficient > 0.4 or <−0.4 with *ADAR*, *ADARB1*, or *ADARB2* were considered significantly associated. Pathway analysis was then conducted using the Reactome Pathway Database (https://reactome.org; accessed on 1 April 2020) to identify overrepresented signaling pathways among the correlated genes. Overrepresentation analysis was based on a hypergeometric distribution.

### 2.6. Analysis of VIM Promoter Methylation Using the UALCAN Web Portal

The UALCAN web portal was used to analyze and compare VIM promoter methylation patterns in non-cancerous tissue and primary tumor samples. The methylation level, ranging from 0 (unmethylated) to 1 (fully methylated) was estimated using the beta-value, which is the ratio of the methylated probe intensity to the sum of methylated and unmethylated probe intensity.

The boxplots were generated using the UALCAN web portal and represented the mean of beta-values. Differentially methylated promoters were identified based on statistical (Student’s *t*-test ≤ 0.05) and biological (methylation level difference (Δβ-value) between the groups equal or higher than 0.05) thresholds.

### 2.7. Gene Group Enrichment Analysis

GSEA was conducted using tools available at www.gsea-msigdb.org; accessed on 15 April 2020. Patients were divided into high- and low-expression groups based on average gene expression values. Full transcriptomic profiles, retrieved from the University of California Santa Cruz database were used.

A signaling pathway or biological process was considered significantly enriched if it met the following criteria: (i) nominal *p*-value < 0.05 and (ii) False Discovery Rate (FDR q-value) < 0.25.

### 2.8. Statistical Analysis

All statistical analyses were performed using GraphPad Prism v.7 (GraphPad Software) and Statistica v.13.1 (StatSoft).

Samples (n = 515) were grouped into low-expression and high-expression categories based on the average expression levels of *ADAR*, *ADARB1*, and *ADARB2*. For tumor type-specific analyses, average gene expression thresholds were calculated separately for each tumor location.

Comparisons of gene expression across cancer types were conducted using one-way ANOVA. Normality of data distribution was tested prior to analysis. For non-normally distributed variables, the Kruskal–Wallis test was applied, followed by Dunn’s post hoc test. *p*-value < 0.05 was considered statistically significant, similarly as described earlier [62].

## 3. Results

### 3.1. Expression of ADAR Family Genes in HNSCC Cell Lines

**Figure 1:**
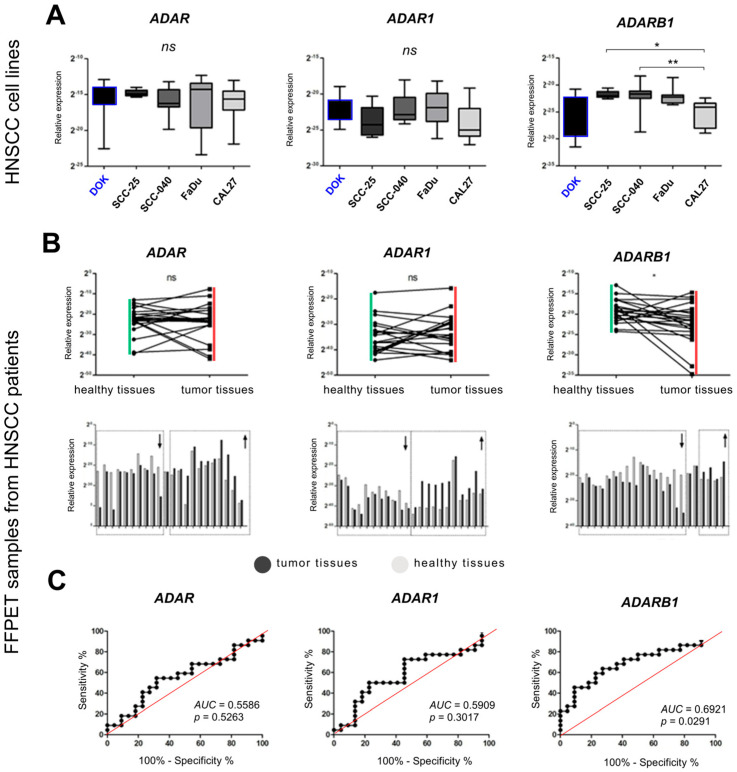
*ADAR*, *ADARB1* and *ADARB2* expression levels in DOK, SCC-25, SCC-40, FaDu and CAL-27 cell lines. *ADAR*, *ADARB1* and *ADARB2* expression levels in normal and cancer tissue from the same patient. (**A**)—the expression levels for all patients; (**B**)—patients stratified into groups with reduced gene expression in tumor tissue (indicated by a downward arrow), identical expression in tumor tissue compared to non-cancerous tissue (not boxed) and increased expression in tumor tissue (marked with an upward arrow); (**C**)—analysis of the quality assessment of the classification of the so-called ROC curve and area under the AUC curve due to *ADAR*, *ADARB1* and *ADARB2* expression levels in non-cancerous and cancer tissue taken from the same patient. *ADARB2* expression level in relation to clinicopathological parameters for FFPET (Formalin-Fixed Paraffin-Embedded Tissue) of patients with HNSCCs; *t*-test; Mann–Whitney test; one-way analysis of variance (ANOVA) with Dunn’s multiple post-test comparisons; *p* < 0.05 was considered significant; * *p* ≤ 0.05, ** *p* ≤ 0.01, ns—no statistical significance.

Quantitative analysis of *ADAR*, *ADARB1*, and *ADARB2* expression was performed in the studied HNSCCs cell lines. No statistically significant differences in expression were observed for *ADAR* and *ADARB1* across the tested lines. In contrast, *ADARB2* expression differed significantly between CAL-27 and SCC-25 (*p* = 0.03), as well as between CAL-27 and SCC-40 (*p* = 0.0012). In both comparisons, *ADARB2* expression was noted to be lower in CAL-27 cells (Figure 1A).

### 3.2. Expression of ADAR, ADARB1, and ADARB2 in Tumor vs. Non-Cancerous Tissues

In clinical samples collected from 10 patients with HNSCC, the expression of all analyzed genes (*ADAR*, *ADARB1*, and *ADARB2*) varied between tumor and adjacent non-cancerous tissues. For *ADAR*, reduced expression in tumor tissue was observed in 45.5% of patients, increased expression in 50%, and no change in 4.5%. *ADARB1* expression showed no consistent trend, being either up- or downregulated in 50% of cases. In contrast, *ADARB2* displayed a distinct profile: 73% of patients showed downregulation in tumors compared to matched non-cancerous tissue, 18% showed upregulation, and 9% presented no change. Despite interpatient variability, statistical analysis revealed that only *ADARB2* expression differed significantly between tumor and normal tissues (*p* = 0.044), with reduced levels in cancer samples. No significant differences were observed for *ADAR* or *ADARB1* (Figure 1B). To explore their diagnostic potential, receiver operating characteristic curve analysis was performed. *ADAR* (AUC = 0.5558, *p* = 0.5263) and *ADARB1* (AUC = 0.5909, *p* = 0.3017) showed limited discriminatory value. In contrast, *ADARB2* achieved an AUC of 0.692 (*p* = 0.0291), indicating moderate discriminatory power and suggesting its potential as a molecular marker for distinguishing malignant from normal tissues (Figure 1C).

### 3.3. Validation of Expression Patterns Using TCGA Data

**Figure 2:**
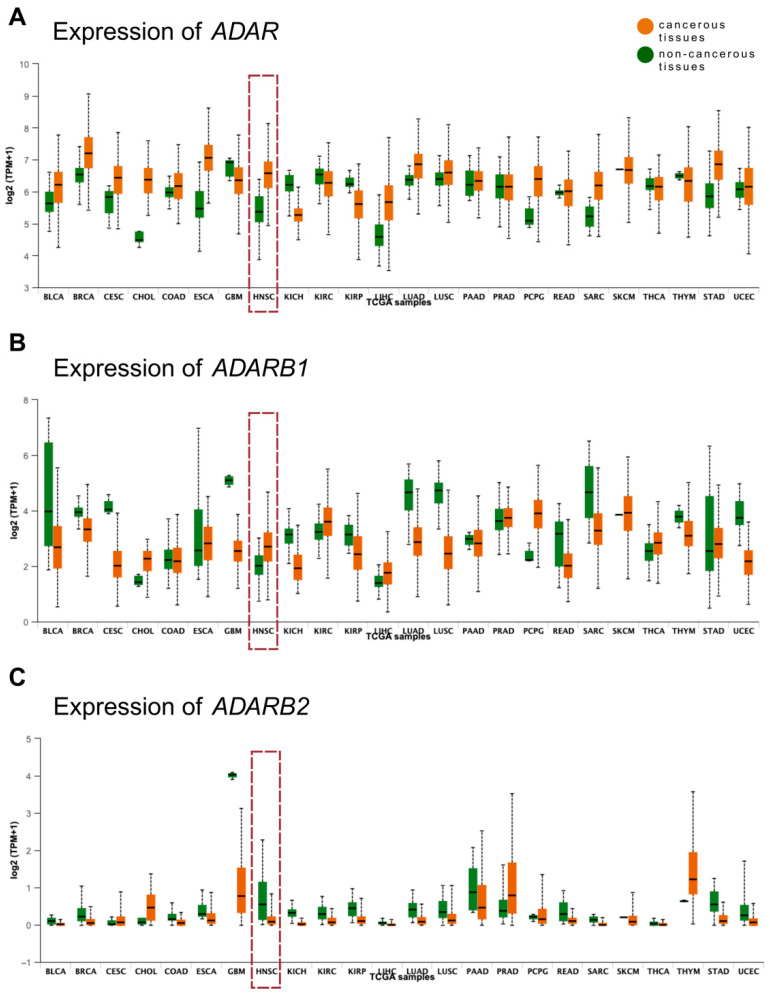
*ADAR* (**A**), *ADARB1* (**B**) and *ADARB2* (**C**) expression levels in 24 tumor types included in the TCGA analysis. Figure retrieved from UALCAN database, modified; the fold change is estimated from the median expression—tumor tissues vs. non-cancerous tissues; TPM—transcripts per million; BLCA—Bladder urothelial carcinoma; BRCA—Breast invasive carcinoma; CESC—Cervical squamous cell carcinoma; CHOL—Cholangiocarcinoma; COAD—Colon adenocarcinoma; ESCA—Esophageal carcinoma; GBM—Glioblastoma multiforme; HNSC—Head and Neck squamous cell carcinoma; KICH—Kidney chromophobe; KIRC—Kidney renal clear cell carcinoma; KIRP—Kidney renal papillary cell carcinoma; LIHC—Liver hepatocellular carcinoma; LUAD—Lung adenocarcinoma; LUSC—Lung squamous cell carcinoma; PAAD—Pancreatic adenocarcinoma; PRAD—Prostate adenocarcinoma; PCPG—Pheochromocytoma and Paraganglioma; READ—Rectum adenocarcinoma; SARC—Sarcoma; SKCM—Skin cutaneous melanoma; THCA—Thyroid carcinoma; THYM—Thymoma; STAD—Stomach adenocarcinoma; UCEC—Uterine corpus endometrial carcinoma.

Based on TCGA clustering data for *ADAR*, *ADARB1*, and *ADARB2*, fold changes in gene expression between tumor and matched non-cancerous tissues were calculated using median expression values. For *ADAR*, expression patterns varied depending on cancer type, showing either upregulation, downregulation, or no significant difference between tumor and normal tissues. The lowest fold change was observed in the kidney chromophobe (KICH; 0.85), while the highest was in cholangiocarcinoma (CHOL; 1.42). In HNSCCs, the fold change was 1.23, indicating elevated *ADAR* expression in tumor tissues compared to non-cancerous controls (Figure 2A). For *ADARB1*, both increased and decreased expressions were observed across cancer types. The lowest fold changes were reported in cervical squamous cell carcinoma and glioblastoma multiforme (GBM; 0.5), whereas the highest was in pheochromocytoma and paraganglioma (PCPG). In HNSCC, the fold change reached 1.34, suggesting higher *ADARB1* expression in tumors relative to normal tissues (Figure 2B). In contrast, *ADARB2* expression was predominantly reduced in cancers, with downregulation observed in approximately 75% of analyzed tumor types. The lowest fold change was recorded in the kidney chromophobe (KICH; 0.05), whereas the highest was in prostate adenocarcinoma (PRAD). In HNSCC, *ADARB2* expression was markedly suppressed in tumors, with a fold change of 0.15 compared to non-cancerous tissues (Figure 2C).

**Figure 3:**
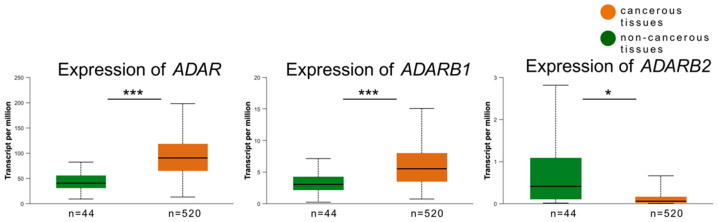
Expression levels of ADAR family genes in HNSCCs tumor versus non-cancerous tissues. Box plots present the transcript per million (TPM) values of *ADAR*, *ADARB1*, and *ADARB2* in tumor tissues (n = 520, orange) and matched non-cancerous tissues (n = 44, green) obtained from TCGA UALCAN datasets. Expression of *ADAR* and *ADARB1* was significantly upregulated in tumor tissues compared to non-cancerous controls (*p* < 0.001), whereas *ADARB2* showed a significantly decreased expression in tumor samples (*p* < 0.05). *p* < 0.05 was considered significant; * *p* ≤ 0.05, *** *p* ≤ 0.001.

Gene expression analysis revealed that *ADAR* and *ADARB1* were significantly upregulated in tumor tissues compared to normal counterparts. The median expression of *ADAR* was 90.376 in tumor tissues versus 40.05 in non-cancerous tissues (*p* < 0.0001). Similarly, *ADARB1* showed higher expression in tumors (median = 5.486) than in normal tissues (median = 3.021, *p* < 0.0001). In contrast, *ADARB2* expression was significantly reduced in tumors, with a median of 0.056 compared to 0.403 in non-cancerous tissues (*p* = 0.04) (Figure 3).

**Figure 4:**
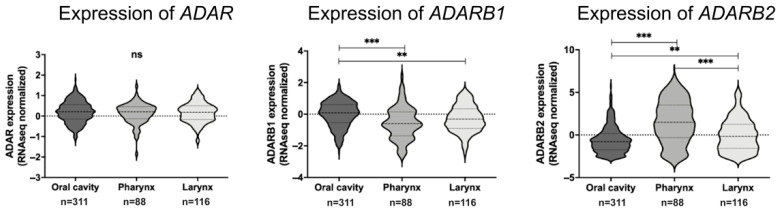
Expression of ADAR family genes across anatomical subsites of HNSCC. Violin plots show normalized RNA-Seq expression levels of *ADAR*, *ADARB1*, and *ADARB2* in tumors located in the oral cavity, pharynx, and larynx. No significant differences were observed in *ADAR* expression between subsites. In contrast, *ADARB1* expression was significantly higher in laryngeal tumors compared to pharyngeal tumors (*p* < 0.001). *ADARB2* expression showed marked variability, with significantly elevated levels in pharyngeal tumors compared to both oral cavity and laryngeal tumors (*p* < 0.01; *p* < 0.001). *p* < 0.05 was considered significant; ns-non significant ** *p* ≤ 0.001, *** *p* ≤ 0.0001.

When stratified by anatomical location, median *ADAR* expression was 0.2103 in oral cavity tumors, 0.2072 in pharyngeal tumors, and 0.1848 in laryngeal tumors, with no significant differences between sites. For *ADARB1*, median expression was 0.0917 in the oral cavity, −0.5945 in the oropharynx, and −0.8961 in the larynx. Expression in the oral cavity was significantly higher than in the oropharynx (*p* < 0.0001). For *ADARB2*, median expression values were −0.778 in the oral cavity, 1.491 in the oropharynx, and −0.1747 in the larynx. Significant differences were observed between the oral cavity and oropharynx, as well as between the larynx and pharynx (*p* < 0.0001). The highest *ADARB2* expression was detected in oropharyngeal tumors (Figure 4).

### 3.4. Promoter Methylation Analysis of ADAR Family Genes

**Figure 5:**
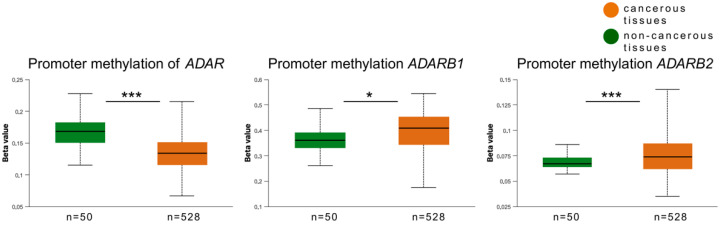
Promoter methylation levels of ADAR family genes in HNSCC. Box plots show promoter methylation (β-values) of *ADAR*, *ADARB1*, and *ADARB2* in tumor tissues (n = 528, orange) and non-cancerous tissues (n = 50, green) from TCGA UALCAN datasets. Tumor samples displayed significantly lower promoter methylation of *ADAR* (*p* < 0.001) and *ADARB2* (*p* < 0.001), while *ADARB1* showed significantly increased promoter methylation compared to non-cancerous tissues (*p* < 0.05). *p* < 0.05 was considered significant; * *p* ≤ 0.05, *** *p* ≤ 0.001.

Promoter methylation analysis showed distinct regulatory patterns among the three genes. For *ADAR*, promoter methylation levels were significantly higher in non-cancerous tissues (median beta value = 0.168) than in tumors (median = 0.134, *p* < 1.3 × 10^−9^). Conversely, *ADARB1* displayed significantly increased promoter methylation in tumors (median = 0.408) compared to normal tissues (median = 0.360, *p* < 0.011). A similar trend was observed for *ADARB2*, where tumors exhibited higher promoter methylation (median = 0.074) than non-cancerous tissues (median = 0.067, *p* < 00001) (Figure 5).

### 3.5. Prognostic Significance of ADAR Family Expression

**Figure 6:**
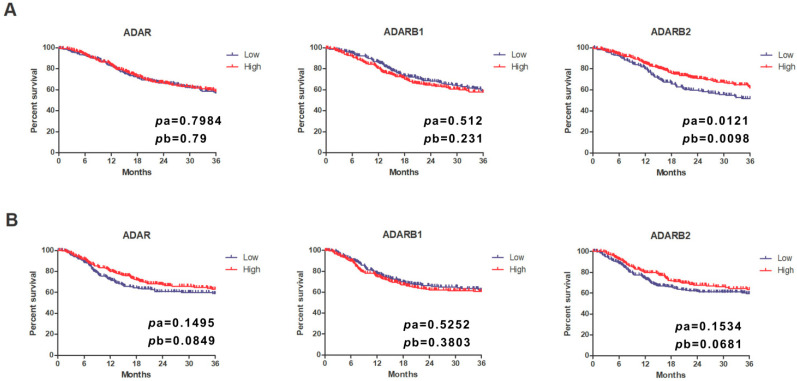
Overall survival (**A**) and progression-free survival (**B**) of patients with head and neck cancers with low and high expression of *ADAR*, *ADARB1* and *ADARB2* genes. The results are presented assuming a 36-month observation period, groups with low and high expression were separated on the basis of the average expression of a given gene; a—log-rank test (Mantel–Cox), b—Gehan-Breslow-Wilcoxon test; *p* < 0.05 was considered statistically significant.

Survival analysis showed that overall survival differed significantly only in relation to *ADARB2* expression. Patients with high *ADARB2* expression demonstrated significantly longer overall survival compared to those with low expression (pa = 0.0121, pb = 0.0098). When evaluating progression-free survival, no statistically significant associations were detected for any of the three genes. However, trends toward significance were observed for *ADAR* (pb = 0.0849) and *ADARB2* (pb = 0.0681), with patients showing high expression of these genes displaying longer PFS (Figure 6).

**Figure 7:**
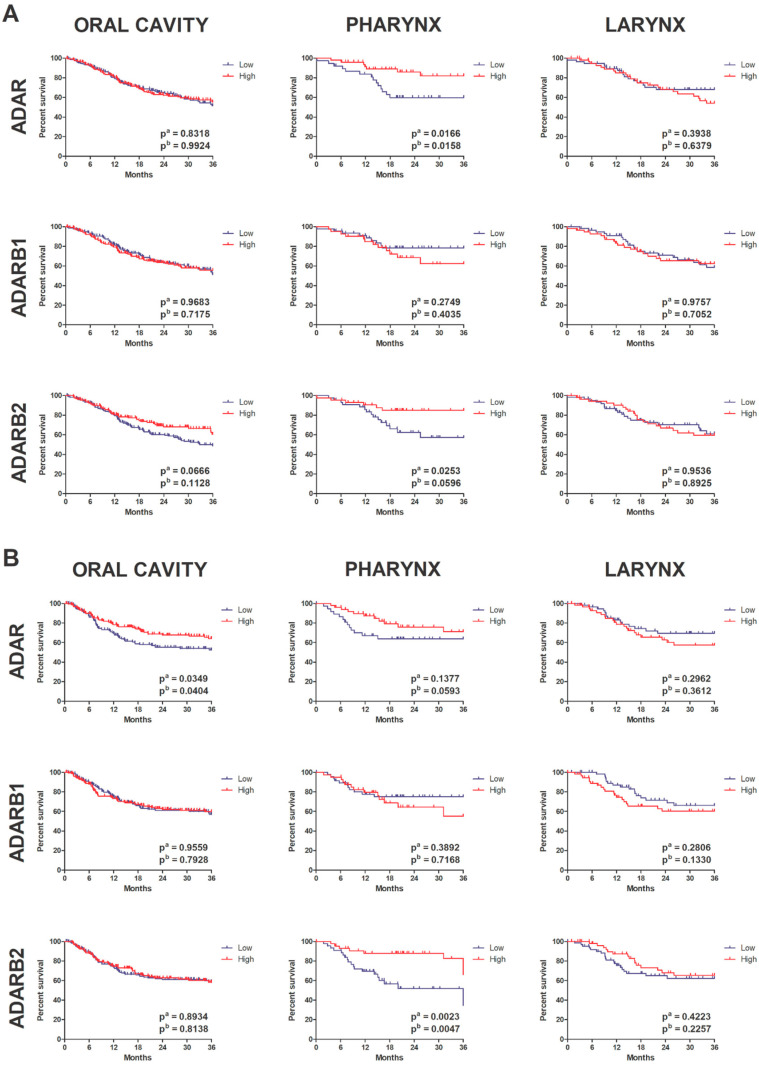
Kaplan–Meier plots illustrate the association between mRNA expression levels of *ADAR*, *ADARB1*, and *ADARB2* with patient survival across three anatomical subsites: oral cavity, pharynx, and larynx. Patients were stratified into high (red) and low (blue) expression groups. Panel (**A**) presents Overall survival (OS) analysis. Panel (**B**) presents Disease-free survival (DFS) analysis. Log-rank test *p*-values are indicated on each plot (pa = univariate; pb = multivariate or secondary test).

To investigate the prognostic value of the ADAR family in HNSCC, we performed Kaplan–Meier survival analyses stratified by anatomical subsite (oral cavity, pharynx, larynx). Patients were divided into high and low expression groups based on median mRNA levels of *ADAR*, *ADARB1*, and *ADARB2*. In the oral cavity subgroup, the expression of *ADAR* and *ADARB2* showed no significant association with overall survival, although a trend toward shorter survival was observed for patients with low *ADARB2* expression. *ADARB1* expression did not influence prognosis in this subgroup. In contrast, disease-free survival analysis revealed that high *ADAR* expression was significantly correlated with unfavorable outcome (pa = 0.0349, pb = 0.0404). In the pharyngeal subgroup, marked differences were observed. High *ADAR* expression was significantly associated with shorter OS (pa = 0.0166, pb = 0.0158). Similarly, elevated *ADARB2* expression correlated with poor OS (pa = 0.0253, pb = 0.0596). Conversely, in DFS analysis, high *ADARB2* expression was strongly associated with improved prognosis (pa = 0.0023, pb = 0.0047), indicating that *ADARB2* may have context-dependent roles in pharyngeal carcinogenesis. No significant prognostic associations were identified for *ADARB1*. In the laryngeal subgroup, expression of *ADAR*, *ADARB1*, and *ADARB2* did not significantly affect either OS or DFS (Figure 7A,B).

### 3.6. Association with Clinicopathological Parameters

Analysis of clinical parameters revealed several significant associations between gene expression and patient characteristics. For *ADAR* expression levels differed by sex, with women showing higher expression than men (0.2690 ± 0.0421 vs. 0.1491 ± 0.0261, *p* = 0.0179). In addition, patients who had undergone cervical lymph node dissection exhibited lower expression compared to those without surgery (0.1471 ± 0.0254 vs. 0.3103 ± 0.0428, *p* = 0.004); (Table 1).

The detailed clinical parameters analyses showed that *ADARB1* expression was also higher in women than in men (0.0664 ± 0.0718 vs. −0.2510 ± 0.0453, *p* = 0.0006). Significantly elevated expression was observed in non-smokers compared to smokers (0.0102 ± 0.0664 vs. −0.2829 ± 0.0484, *p* = 0.0005), and in patients with lower tumor dedifferentiation grades (G1–G2 vs. G3–G4: −0.1116 ± 0.0440 vs. −0.2643 ± 0.0776, *p* = 0.0379). Furthermore, expression was also significantly higher in HPV-negative patients compared to HPV-positive ones (0.0035 ± 0.0959 vs. −0.7256 ± 0.1516, *p* < 0.001); (Table 2).

In the case of *ADARB2*, the different pattern was noted. Men exhibited higher expression compared to women (0.0858 ± 0.1077 vs. −0.5725 ± 0.1234, *p* = 0.0077), and alcohol consumers had higher expression than non-drinkers (0.0401 ± 0.1084 vs. −0.3386 ± 0.1500, *p* = 0.0298). Higher expression was characteristic of tumors with high dedifferentiation (G3–G4 vs. G1–G2: 0.5701 ± 0.2075 vs. −0.4076 ± 0.0865, *p* = 0.0002), as well as smaller tumor volumes (T1–T2 vs. T3–T4: 0.2752 ± 0.1531 vs. −0.2685 ± 0.1063, *p* = 0.0035). Patients who had undergone cervical lymphadenectomy showed reduced expression compared to those without surgery (−0.2479 ± 0.0899 vs. 0.5848 ± 0.2424, *p* = 0.0054). In contrast to *ADARB1*, HPV-positive patients had significantly higher *ADARB2* expression compared to HPV-negative ones (2.084 ± 0.3382 vs. −0.1426 ± 0.1814, *p* < 0.0001); (Table 3).

### 3.7. Correlation and Pathway Enrichment Analyses

**Figure 8:**
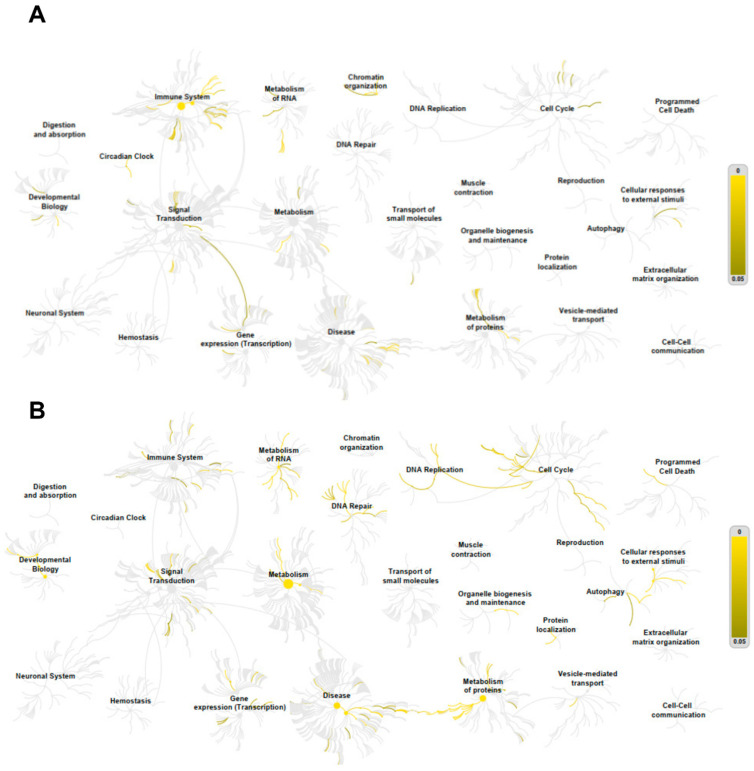
Positive (**A**) and negative (**B**) *ADAR* correlation with genes involved in important cellular processes. Only genes with a Spearman correlation > 0.4, <−0.4 and *p* < 0.05 were indicated in the REACTOME pathway analysis as yellow lanes in the graphs.

Pathway analyses were performed using the Reactome database for genes positively and negatively correlated with *ADAR*, *ADARB1*, and *ADARB2* expression. For *ADAR*, positively correlated genes were enriched in pathways related to the immune system, cell cycle, RNA metabolism, signal transduction, and protein metabolism. Notable pathways included interferon-α/β signaling (31/186), cytokine signaling (90/1312), interferon-mediated antiviral mechanisms (20/94), chromatin organization (26/256), IL-6 (7/17) and IL-27 (5/13) signaling, and protein sumoylation (7/43) (Figure 8A). In contrast, negatively correlated genes were enriched in pathways linked to RNA and protein metabolism, translation, and cell cycle regulation, such as nonsense-mediated mRNA decay (56/101), 40S ribosome subunit formation (56/106), translation initiation (56/130), rRNA processing (61/245), and elongation of the polypeptide chain (57/102) (Figure 8B).

**Figure 9:**
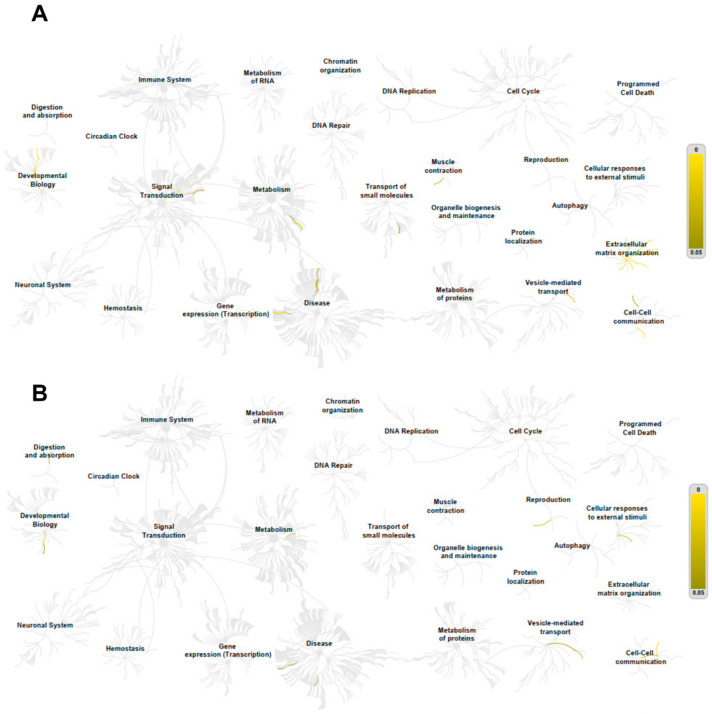
Positive (**A**) and negative (**B**) *ADARB1* correlation with genes involved in important cellular processes. Only genes with a Spearman correlation > 0.4, <−0.4 and *p* < 0.05 were indicated in the REACTOME pathway analysis as yellow lanes in the graphs.

In case of *ADARB1*, positively correlated genes were mainly associated with extracellular matrix organization and intercellular communication, including collagen trimerization (8/44), biosynthesis and modification of collagen (9/76), collagen degradation (8/69), extracellular matrix organization (12/329), integrin interactions (7/86), and laminin interactions (4/31) (Figure 9A). Negatively correlated genes were linked to cell junction organization, pluripotency regulation, hyaluronan metabolism, and branched-chain amino acid catabolism (Figure 9B).

**Figure 10:**
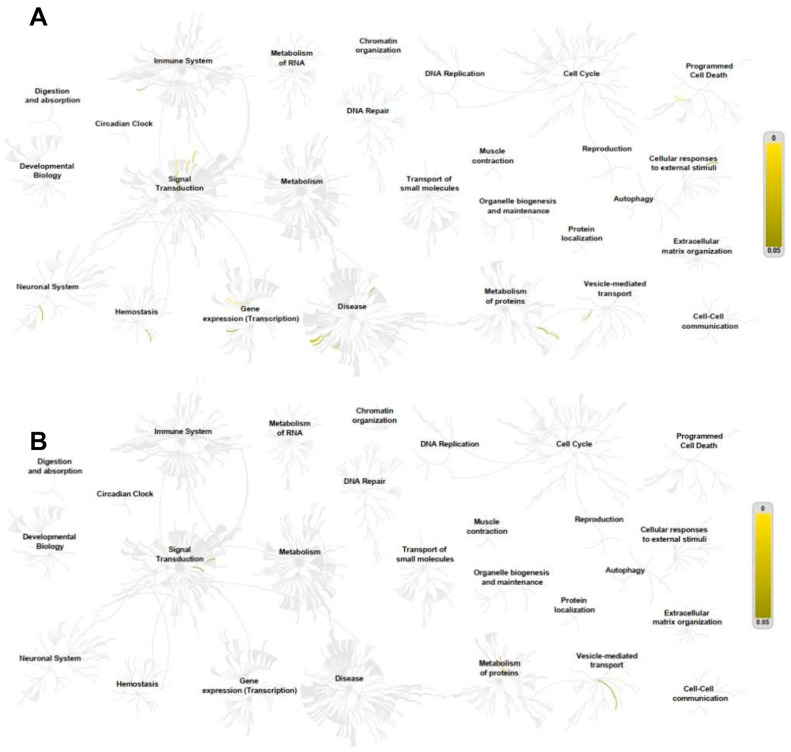
Positive (**A**) and negative (**B**) *ADARB2* correlation with genes involved in important cellular processes. Only genes with a Spearman correlation > 0.4, <−0.4 and *p* < 0.05 were indicated in the REACTOME pathway analysis as yellow lanes in the graphs.

For *ADARB2*, positively correlated genes were enriched in pathways related to transcriptional regulation and apoptosis, including nuclear receptor transcription (6/86), NOTCH3 signaling (3/63), and inactivation of Bcl-2 anti-apoptotic proteins via BH3-only proteins (2/11). Negatively correlated genes were enriched in small GTPase pathways, including RAB protein geranylgeranylation (1/65), GDP-to-GTP activation (1/90), and Rho GTPase cycling (1/141) (Figure 10).

### 3.8. Gene Set Enrichment Analysis

**Figure 11:**
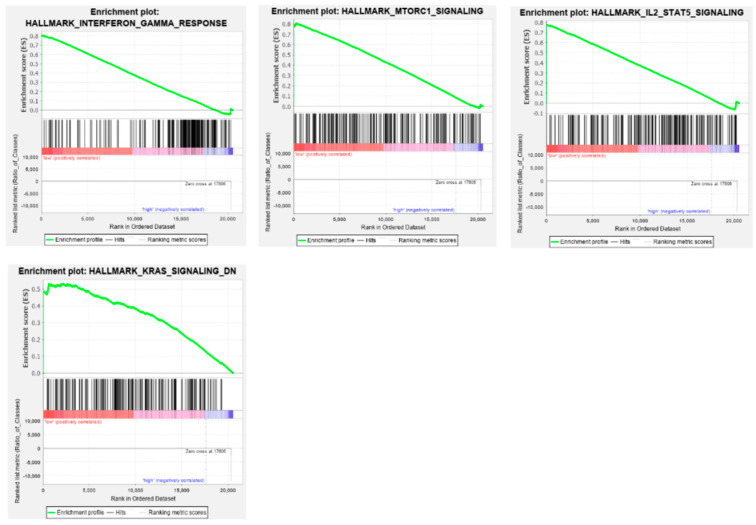
Gene Set Enrichment Analysis (GSEA) of *ADAR* expression in HNSCC. Normalized enrichment scores (NES) for MSigDB Hallmark gene sets significantly enriched in patients with low (red) or high (blue) expression of ADAR. Only pathways with a nominal *p*-value ≤ 0.05 and a false discovery rate (FDR) ≤ 0.25 are presented.

GSEA further highlighted biological differences between patients with high and low expression of the three ADAR family genes. For ADAR, patients with low expression demonstrated enrichment of several cancer-related pathways, including interferon-γ response (*p* = 0.009, FDR = 0.091), mTORC1 signaling (*p* = 0.011, FDR = 0.204), and IL-2/STAT5 signaling (*p* = 0.018, FDR = 0.127), together with downregulation of KRAS signaling (*p* = 0.047, FDR = 0.200) (Figure 11).

**Figure 12:**
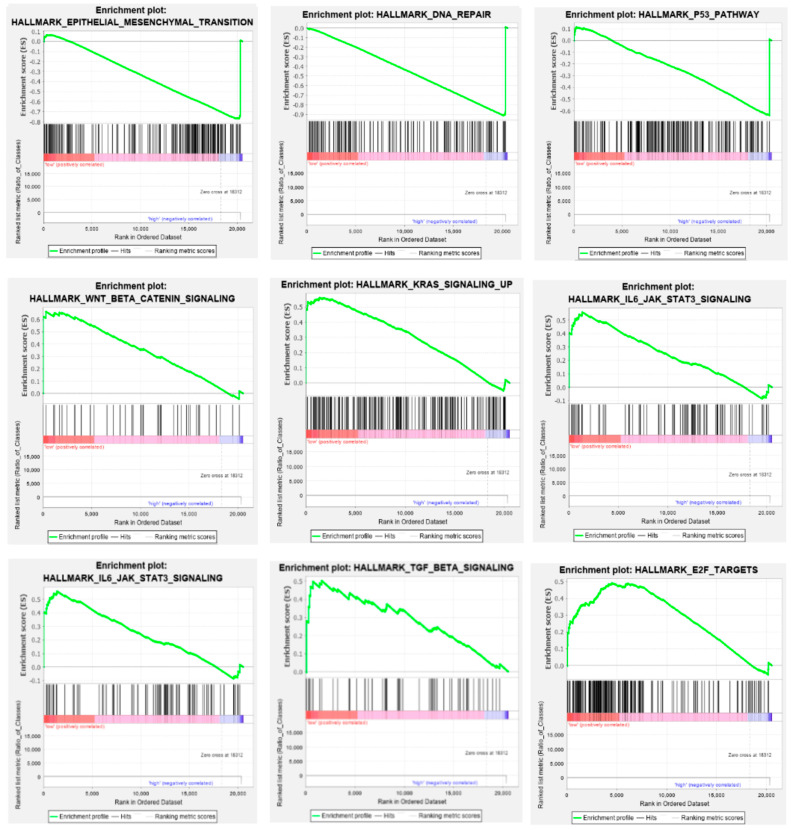
Gene Set Enrichment Analysis (GSEA) of *ADARB1* expression in HNSCC. Normalized enrichment scores (NES) for MSigDB Hallmark gene sets significantly enriched in patients with low (red) or high (blue) expression of ADAR. Only pathways with a nominal *p*-value ≤ 0.05 and a false discovery rate (FDR) ≤ 0.25 are presented.

For *ADARB1*, enrichment was observed in both high- and low-expression groups, suggesting context-dependent functions of this enzyme. High expression was associated with activation of pathways related to genomic stability and tumor suppression, including epithelial–mesenchymal transition (*p* = 0.020, FDR = 0.191), DNA repair (*p* = 0.022, FDR = 0.164), and the p53 pathway (*p* = 0.036, FDR = 0.120). In contrast, low expression correlated with enrichment of oncogenic and inflammatory signaling cascades, such as Wnt/β-catenin signaling (*p* = 0.011, FDR = 0.127), KRAS activation (*p* = 0.023, FDR = 0.139), IL-6/STAT3 signaling (*p* = 0.025, FDR = 0.125), TGFB1 response (*p* = 0.027, FDR = 0.129), IL-2/STAT5 signaling (*p* = 0.039, FDR = 0.142), and E2F-mediated cell cycle control (*p* = 0.046, FDR = 0.145) (Figure 12).

**Figure 13:**
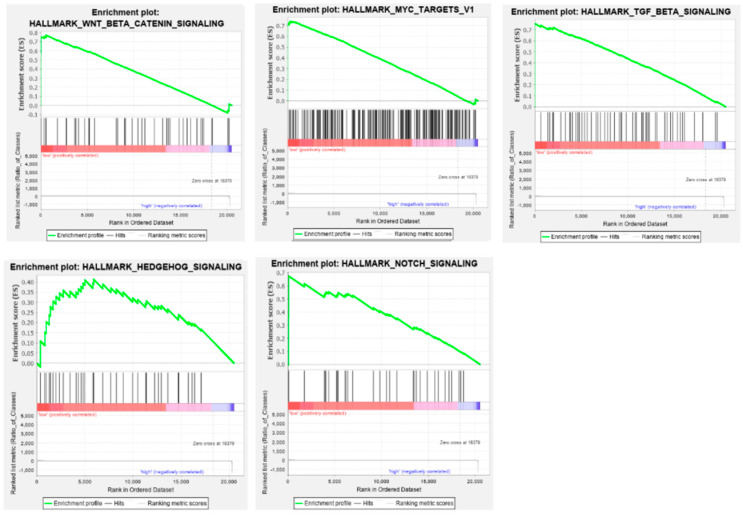
Gene Set Enrichment Analysis (GSEA) of *ADARB2* expression in HNSCC. Normalized enrichment scores (NES) for MSigDB Hallmark gene sets significantly enriched in patients with low (red) or high (blue) expression of ADAR. Only pathways with a nominal *p*-value ≤ 0.05 and a false discovery rate (FDR) ≤ 0.25 are presented.

For *ADARB2*, pathway enrichment was detected only in patients with low expression, pointing to a loss of regulatory functions in this context. These pathways included Wnt/β-catenin signaling (*p* = 0.006, FDR = 0.174), MYC target regulation (*p* = 0.009, FDR = 0.125), TGFB1 response (*p* = 0.009, FDR = 0.156), Hedgehog signaling (*p* = 0.010, FDR = 0.206), and Notch signaling (*p* = 0.036, FDR = 0.233) (Figure 13).

## 4. Discussion

This study provides the first in-depth analysis of the expression and clinical significance of the ADAR family genes in HNSCCs. While ADAR1 and ADAR2 have been implicated in cancer biology across multiple tumor types, the role of *ADARB2* remains poorly understood [65]. Our findings reveal that *ADARB2* expression is consistently reduced in HNSCC tissues compared with normal mucosa and that patients with higher *ADARB2* expression exhibit significantly improved overall survival. These results highlight *ADARB2* as a candidate diagnostic and prognostic biomarker in this malignancy.

In silico analyses of TCGA cohorts confirmed differential expression of all three ADAR family members, with *ADAR* (*ADAR1*) and *ADARB1* (*ADAR2*) being significantly upregulated in tumor tissues, while *ADARB2* (*ADAR3*) was markedly downregulated. Importantly, our validation using paired tumor and adjacent non-cancerous tissues confirmed this downregulation for *ADARB2* but not for *ADAR* or *ADARB1*, suggesting that *ADARB2* may represent the most robust biomarker candidate in this context. This observation is particularly relevant, as *ADARB2* expression allowed clear discrimination between malignant and non-malignant tissues, with ROC analysis indicating promising diagnostic performance. The results obtained by Zhang et al., who observed that high levels of *ADARB2* expression are associated with a better prognosis in patients with low-grade glioma, and their multivariate analysis suggests that it is an independent prognostic factor [59]. Similarly, Cesarini et al. demonstrated that in glioblastoma, it is not the *ADAR2* transcript level but rather the protein abundance that correlates with patient survival, with higher *ADAR2* expression being particularly associated with prolonged overall survival [66].

Our findings also need to be interpreted in the context of recent studies on ADAR1 in oral squamous cell carcinoma (OSCC). In one report based on TCGA and immunohistochemistry of 46 OSCC cases, nuclear ADAR1 expression correlated with higher histological grade, while cytoplasmic expression emerged as an independent protective prognostic factor [67]. Another study demonstrated that ADAR1 is overexpressed in OSCC tissues and cell lines, where it enhances stemness and epithelial–mesenchymal transition and interacts with Dicer to promote the maturation of oncogenic microRNAs, ultimately associating with poor prognosis [68]. Taken together, these data highlight the multifaceted and context-dependent functions of ADAR1 in head and neck carcinogenesis. While ADAR1 appears to act as an oncogenic driver through RNA-editing–dependent and independent mechanisms, ADARB2 in contrast emerges as a potential tumor suppressor, with its loss linked to enrichment of oncogenic pathways in our GSEA analyses. This functional divergence within the ADAR family underscores the complexity of RNA-editing enzymes in cancer biology.

Interestingly, analyzes of the overall survival time, which were performed for patients divided into groups according to the location of the tumor, showed that this dependence occurs mainly in those patients in whom the tumor is located in the throat. A significantly longer overall survival was also observed in patients with tumors at this location who exhibited high *ADAR* expression. Although *ADAR* is frequently described in the literature as an oncogene [41,50,58], evidence suggests that its role in carcinogenesis is more complex. Not only increased *ADAR* expression but also gene silencing or deletion may contribute to tumor development, as reported in malignant melanoma and breast cancer [69]. In the latter, the ADAR1 enzyme modifies the GABRA3 receptor, which in breast cancer promotes activation of the Akt pathway, leading to enhanced cell motility, proliferation, and metastasis. ADAR1-dependent editing of this receptor reduces its surface expression, thereby diminishing the metastatic potential of tumor cells.

The observed differences in overall survival according to tumor location may also reflect distinct molecular backgrounds underlying HNSCCs. Despite sharing the same histological classification, the mechanisms of carcinogenesis vary between anatomical sites. For example, Shiga et al. demonstrated that in laryngeal and pharyngeal cancers, tumor formation is driven by loss of suppressor gene function through allelic deletion, whereas in oral cavity cancers the same suppressor genes are inactivated predominantly by promoter methylation [70].

Real-time PCR analysis of *ADAR*, *ADARB1* and *ADARB2* expression showed that of the 76 patient samples, only 26% had ADAR amplification and 15% *ADARB1*. This suggests that the promoters of these genes are likely to be methylated due to their activity, leading to a change in RNA expression levels [71]. On the other hand, *ADARB2*, which lacks catalytic properties, was amplified in all tested samples. It should be noted that the study utilized RNA isolated from archival material (paraffin blocks), which may have influenced the obtained results due to modifications and progressive degradation of RNA [72,73]. Performing an analysis of promoter methylation in these samples would unequivocally determine whether the observed changes are caused by these epigenetic modifications. This is crucial because the data obtained from the TCGA database indicate reduced methylation of the promoter of the gene encoding ADAR and increased methylation in the case of *ADARB1* and *ADARB2* in cancer tissue compared to non-cancerous tissue.

Due to the lack of sufficient data for statistical analysis of *ADAR* and *ADARB1*, such analyses were performed only for *ADARB2*. In the TCGA dataset, the highest *ADARB2* expression was observed in pharyngeal tumors and the lowest in oral cavity tumors. In contrast, analysis of patient samples from the Greater Poland Oncology Centre revealed the highest expression in oral cavity tumors and the lowest in laryngeal tumors. These discrepancies may reflect population-specific differences between the TCGA cohort and the clinical population being studied. A gradual, though not statistically significant, decrease in *ADARB2* expression was also noted with increasing tumor volume and higher histological grade. This suggests that reduced *ADARB2* expression, and consequently increased activity of the ADAR3 enzyme, may be associated with a more unfavorable clinical outcome. The location-dependent differences in expression identified by in silico analysis were not fully reproduced in patient-derived tumor samples. While *ADARB2* expression varied by anatomical site, the distribution patterns differed between TCGA data and clinical samples, again likely reflecting population differences.

Expression analysis in cell lines showed no significant differences for *ADAR* and *ADARB1*, whereas *ADARB2* expression varied between specific lines. Interestingly, no differences were found between the DOK line (dysplastic oral keratinocytes, used as control) and other lines. Instead, significant differences were observed between SSC-25 and CAL-27 as well as SCC-40 and CAL-27. The biological relevance of these findings is difficult to interpret, particularly given the absence of published data on *ADARB2* expression in head and neck cancer cell lines.

Analysis of the level of *ADARB2* gene expression depending on clinical data showed differences for such factors as, e.g., alcohol consumption, sex, cervical lymphadenectomy, and HPV infection status. The results suggest that reduced expression of this gene, and thus a better prognosis, is associated with no alcohol consumption, female gender, lymph node dissection, and HPV negative status. These results are surprising, taking into account mainly the last of the studied factors, because it has been shown that HPV infection results in a better clinical picture of the disease and increased susceptibility to chemo- and radiotherapy [74]. However, it should be noted that this analysis is univariate and does not fully capture the complexity of the relationship between HPV infection and disease progression, but rather compares groups based on the presence or absence of infection due to the level of gene expression.

In this study, the phenotype of patients in groups depending on the low and high level of expression of the studied genes was also analyzed using the GSEA tool, which compares the analyzed groups with the known profile of gene expression from a given pathway or process. Among the signaling pathways that are enriched in patients with low expression of the *ADARB2* gene compared to the group with high expression, changes in the Wnt/β-catenin pathway, whose excessive activation is associated with the development and progression of HNSCCs have been observed [75]. Under homeostasis, the activity of this signaling pathway is kept low because the genes associated with it determine cell differentiation and proliferation. In the case of excessive activation, β-catenin accumulates in the cytoplasm and is then transferred to the cell nucleus, where it activates transcription factors, consequently promoting the expression of such proto-oncogenes as MYC or cyclin D1 [76]. Enrichment of target genes for the MYC transcription factor was also noted, confirming the possibility of excessive activation of this oncogenic pathway. The results described above suggest the existence of a mechanism among patients with high ADARB2 expression that prevents overexpression of genes belonging to the Wnt/β-catenin pathway and MYC target genes (it should be remembered that both compared groups include oncology patients; it is not a comparison to healthy patients), which may be directly related to a better prognosis.

In the group of patients with reduced expression of the ADARB2 gene, characterized by a shorter overall survival, signaling pathways such as Notch, Hedgehog and genes activated by TGF-β1 (transforming growth factor β1) are also enriched. According to the literature, the Notch signaling pathway is often overexpressed in HNSCC patients, and the level of gene expression of this pathway is significantly associated with the clinical progression of the disease [77]. It has also been shown that this pathway is involved in the process of epithelial–mesenchymal transition, but the NOTCH1 gene itself may act both as an oncogene and as a tumor suppressor [78]. The Hedgehog pathway, on the other hand, is involved in embryonic development, and in the adult organism, the genes of this pathway are active only under certain conditions, such as during wound healing, or are not activated at all [79]. The Hedgehog pathway is directly related to the development of carcinogenesis and the progression of tumor cell dedifferentiation [80]. The last of the analyzed signaling pathways combines genes activated by the cytokine TGF-β1, which include, among others, various growth factors and other signaling proteins. In the case of non-cancerous epithelial cells, this cytokine has suppressor properties; however, with the progression of the neoplastic process, it may become an oncogenic factor [81].

This study has some limitations. The number of matched tumor and non-cancerous tissues was limited, which reduced statistical power and prevented multivariate survival analyses. The use of archival FFPE material may also have affected RNA quality despite rigorous controls. Our analyses focused on expression and bioinformatics without functional validation, so the causal role of *ADARB2* in HNSCC remains to be established. Discrepancies between TCGA and our cohort likely reflect population differences, and future studies in larger, independent cohorts are needed. Finally, we examined transcript levels only, and future work should address protein abundance and subcellular localization of ADAR family members.

In light of the above evidence, a hypothesis can be formulated regarding the potential use of the ADARB2 gene as a marker for neoplastic tissues in head and neck cancers. This gene is significantly reduced in tumor tissue expression compared to non-cancerous tissue. Oncological patients who are characterized by high expression of the ADARB2 gene also show significantly longer overall survival, which may translate into the use of its expression level as a prognostic factor. Enriched signaling pathways in patients with reduced expression of the discussed gene could be a potential point of reference for targeted therapies.

## 5. Conclusions

Our study demonstrates that the ADAR family, particularly *ADARB2*, plays a critical role in the biology of HNSCCs. *ADARB2* expression is significantly reduced in tumor tissues compared with adjacent normal mucosa. Its discriminatory value suggests potential utility as a molecular biomarker for distinguishing malignant from non-malignant tissues. Patients with high *ADARB2* expression exhibit significantly longer overall survival, indicating its value as a prognostic factor. Moreover, low *ADARB2* expression is associated with the enrichment of oncogenic pathways such as Wnt/β-catenin, Notch, Hedgehog, and TGF-β1 signaling, which may contribute to aggressive tumor behavior.

The main findings highlight *ADARB2* as a promising candidate for integration into molecular diagnostics and risk stratification of HNSCC patients. Further studies in larger, HPV-stratified cohorts are warranted to validate its prognostic value and explore its therapeutic relevance.

## Figures and Tables

**Table 1:** Association between *ADAR* expression and clinicopathological features in patients with HNSCC. The table presents mean expression levels of *ADAR* (±SEM) in relation to patient demographics (age, sex), lifestyle factors (alcohol consumption, smoking), and tumor characteristics (differentiation grade, tumor size, lymph node involvement, perineural invasion, clinical stage, cervical lymph node dissection, and HPV infection status). *t*-test; Mann–Whitney test; *p* < 0.05 was considered significant.

Parameter		Mean ± SEM	*p*
Age	<60	0.144 ± 0.034	0.1374
	≥60	0.211 ± 0.029	
Sex	Female	0.269 ± 0.042	0.0179
	Male	0.149 ± 0.021	
Alcohol consumption	Yes	0.186 ± 0.029	0.9523
	No	0.189 ± 0.036	
Smoking	Yes	0.158 ± 0.029	0.1645
	No	0.223 ± 0.036	
Degree of tumor cell differentiation	G1 + G2	0.182 ± 0.026	0.7805
	G3 + G4	0.196 ± 0.047	
Tumor size	T1 + T2	0.197 ± 0.034	0.6659
	T3 + T4	0.176 ± 0.03	
Presence of cancer cells in lymph nodes	N0	0.194 ± 0.031	0.6965
	N1 + N2 + N3	0.176 ± 0.034	
Perineural space invasion	Yes	0.215 ± 0.039	0.1411
	No	0.135 ± 0.038	
Degree of cancer cell spread	I + II	0.185 ± 0.044	0.9648
	III + IV	0.183 ± 0.026	
Cervical lymph node dissection	Yes	0.147 ± 0.025	0.0043
	No	0.31 ± 0.043	
HPV infection status	Positive	0.088 ± 0.079	0.4388
	Negative	0.175 ± 0.068	

**Table 2:** Association between *ADARB1* expression and clinicopathological features in patients with HNSCC. The table presents mean expression levels of ADARB1 (±SEM) in relation to patient demographics (age, sex), lifestyle factors (alcohol consumption, smoking), and tumor characteristics (differentiation grade, tumor size, lymph node involvement, perineural invasion, clinical stage, cervical lymph node dissection, and HPV infection status). *t*-test; Mann–Whitney test; *p* < 0.05 was considered significant.

Parameter		Mean ± SEM	*p*
Age	<60	−0.195 ± 0.057	0.6462
	≥60	−0.146 ± 0.053	
Sex	Female	0.066 ± 0.072	0.0006
	Male	−0.251 ± 0.045	
Alcohol consumption	Yes	−0.186 ± 0.046	0.3195
	No	−0.124 ± 0.077	
Smoking	Yes	−0.283 ± 0.048	0.0005
	No	0.01 ± 0.066	
Degree of tumor cell differentiation	G1 + G2	−0.112 ± 0.044	0.0379
	G3 + G4	−0.264 ± 0.078	
Tumor size	T1 + T2	−0.221 ± 0.068	0.342
	T3 + T4	−0.134 ± 0.048	
Presence of cancer cells in lymph nodes	N0	−0.117 ± 0.057	0.2001
	N1 + N2 + N3	−0.211 ± 0.055	
Perineural space invasion	Yes	−0.08 ± 0.064	0.1919
	No	−0.221 ± 0.068	
Degree of cancer cell spread	I + II	−0.147 ± 0.081	0.7109
	III + IV	−0.174 ± 0.045	
Cervical lymph node dissection	Yes	−0.148 ± 0.042	0.3653
	No	−0.247 ± 0.104	
HPV infection status	Positive	−0.726 ± 0.152	<0.0001
	Negative	0.004 ± 0.096	

**Table 3:** Association between *ADARB2* expression and clinicopathological features in patients with HNSCC. The table presents mean expression levels of ADARB2 (±SEM) in relation to patient demographics (age, sex), lifestyle factors (alcohol consumption, smoking), and tumor characteristics (differentiation grade, tumor size, lymph node involvement, perineural invasion, clinical stage, cervical lymph node dissection, and HPV infection status). *t*-test; Mann–Whitney test; *p* < 0.05 was considered significant.

Parameter		Mean ± SEM	*p*
Age	<60	−0.064 ± 0.139	0.613
	≥60	−0.101 ± 0.11	
Sex	Female	−0.573 ± 0.123	0.0077
	Male	0.086 ± 0.108	
Alcohol consumption	Yes	0.04 ± 0.108	0.0298
	No	−0.339 ± 0.15	
Smoking	Yes	−0.01 ± 0.109	0.1097
	No	−0.2 ± 0.149	
Degree of tumor cell differentiation	G1 + G2	−0.408 ± 0.087	0.0002
	G3 + G4	0.57 ± 0.208	
Tumor size	T1 + T2	−0.276 ± 0.153	0.0035
	T3 + T4	−0.269 ± 0.106	
Presence of cancer cells in lymph nodes	N0	−0.288 ± 0.114	0.099
	N1 + N2 + N3	0.132 ± 0.136	
Perineural space invasion	Yes	−0.533 ± 0.109	0.1327
	No	−0.048 ± 0.146	
Degree of cancer cell spread	I + II	−0.048 ± 0.18	0.791
	III + IV	−0.07 ± 0.101	
Cervical lymph node dissection	Yes	−0.248 ± 0.09	0.0054
	No	0.585 ± 0.242	
HPV infection status	Positive	2.084 ± 0.338	<0.0001
	Negative	−0.143 ± 0.181	

## Data Availability

The datasets used and/or analyzed during the current study are available from the corresponding author on reasonable request. Raw data are available on the TCGA database. The required data will be provided upon request to the respective individual and/or are available in the TCGA database.
